# Incidence, management and outcome of Hepatic Veno-Occlusive disease /Sinusoidal Obstruction Syndrome after hematopoietic stem cell transplant in Thalassemia major patients: A prospective study of Pakistani BMT

**DOI:** 10.12669/pjms.40.3.7901

**Published:** 2024

**Authors:** Haider Nisar, Memoona Khan, Tariq Azam Khattak, Tariq Ghafoor, Qamar Un Nisa Chaudhry

**Affiliations:** 1 Dr. Haider Nisar, Consultant Pediatrician, Resident Clinical Hematology, Armed Forces Bone Marrow Transplant Center, National Institute of Bone Marrow Transplant Center, Rawalpindi, Punjab, Pakistan; 2 Dr. Memoona Khan, Consultant Hematologist, Armed Forces Bone Marrow Transplant Center, National Institute of Bone Marrow Transplant Center, Rawalpindi, Punjab, Pakistan; 3 Dr. Tariq Azam Khattak, Consultant Pediatrician and BMT Physician, Armed Forces Bone Marrow Transplant Center, National Institute of Bone Marrow Transplant Center, Rawalpindi, Punjab, Pakistan; 4 Dr. Tariq Ghafoor, Armed Forces Bone Marrow Transplant Center, National Institute of Bone Marrow Transplant Center, Rawalpindi, Punjab, Pakistan; 5 Dr. Qamar Un Nisa Chaudhry, Consultant BMT Physician, Director/Commandant, Armed Forces Bone Marrow Transplant Center, National Institute of Bone Marrow Transplant Center, Rawalpindi, Punjab, Pakistan

**Keywords:** Beta Thalassemia major, Hepatic veno-occlusive disease, Sinusoidal obstruction syndrome

## Abstract

**Objectives:**

Hepatic Veno occlusive disease (VOD), also known as sinusoidal obstruction syndrome (VOD/SOS), is a post-transplant life threatening complication. In this study, we aimed to discuss the incidence, management and outcome of VOD in post allogenic transplant patients of beta thalassemia major (BTM).

**Methods:**

A prospective study was conducted in Armed Forces Bone Marrow Transplant Center, between 2001-2022. A total of 385 fully Human Leucocyte Antigen (HLA) matched BTM patients, with Ursodeoxycholic acid for prophylaxis, were included in the study. Incidence of VOD was calculated through cumulative incidence estimates. Chi square test and Mann Whitney test were used to compare discrete and continuous variables respectively. VOD was diagnosed and graded according to European Society for Blood and Marrow Transplantation EBMT Pediatric diagnostic criteria. Risk factors for VOD were grouped as recipient, transplant and donor related. Univariate analysis was performed by log-rank test. All patients who developed VOD were managed primarily with fluid restriction and strict input output monitoring. Statistical analyses were performed using SPSS v 25.0.

**Results:**

Out of 385 transplant patients, forty developed VOD. Median time from date of transplant till onset of VOD was 14 days (range 6-30). Cumulative incidence of all grade VOD was 10.39% (95% CI, 7-14). Eleven out of 40 patients who developed VOD died. Cumulative incidence of Transplant related mortality (TRM) for patients with and VOD was 20.5% (95% CI, 16.6-25.1) vs 27.5% (95% CI, 16.1-42) (p value 0.318) respectively. Among risk factors, age of recipient and fibrosis (p value of 0.04 and 0.000 respectively) were found to be significantly associated with VOD.

**Conclusions:**

Careful selection of transplant candidates before transplant can help reduce the incidence of VOD.

## INTRODUCTION

Pakistan bears the largest burden of thalassemia affecting 5-7% of its population with approximately 5000 thalassemic children born annually.^1^ Although various management approaches including regular transfusions with chelation and splenectomy have been adapted to alleviate the suffering of thalassemics, allogeneic Hematopoietc Stem Cell Transplant (HSCT) is the only curative option for the disorder with survival rates as high as 90%.^2^,^3^ Hepatic veno-occlusive disease/ Sinusoidal Obstruction Syndrome (VOD/SOS) remains a potentially life-threatening complication in recipients of HSCT all over the world.^4^-^7^ Underlying pathophysiology involves sinusoidal endothelial injury leading to extravasation of leukocytes and other debris with consequent occlusion of microcirculation and development of Multi organ dysfunction.^6^,^7^ While International Blood and Marrow Transplant Research (CIBMTR) reports VOD/SOS incidence of 4.9% in post Allogeneic-HSCT patients^8^, reported incidence of VOD/SOS in BTM patients varies from 6.1% to 33%.^9^,^10^ Keeping in view the variable presentation of VOD/SOS, and significant association of the diagnostic criteria with outcome, an updated criteria has been established by European Society for Blood and Marrow Transplantation (EBMT) for diagnosis and severity assessment of VOD/SOS in adults and children.^8^ Several studies emphasize upon significance of early intervention and preemptive treatment strategies to improve outcome in VOD/SOS. Some of the potential risk factors reported in literature include Transplantation-Related: prior liver disease, busulfan (Bu) and cyclophosphamide (Cy) containing conditioning regimen, prophylactic use of calcineurin inhibitors and Patient-Related: age group, Pesaro classification, ferritin and fibrosis.^8^-^11^

We, hereby, report the incidence, risk factors and outcome of this potentially fatal complication in this prospective study in 385 BTM patients.

## METHODS

This is a single center prospective study of BTM patients enrolled consecutively undergoing matched related HSCT at Armed Forces Bone marrow transplant center/National Institute of Bone Marrow Transplant (AFBMTC/NIBMT) between October 2001 to December 2022We enrolled a total of 385 BTM patients. DNA based low/intermediate resolution typing for HLA Class-I and II antigens was determined and all patients were 6/6 antigen matched with the donor, either sibling or parents. All patients had Eastern Cooperative Oncology Group (ECOG) performance score of zero. Baseline values of bilirubin, liver size and body weight were determined for all the patients before HSCT.

### Ethical Approval

Approval was obtained from the hospital ethics committee for conducting the study (Ref: IRB-008/AFBMTC/Approval /2023). Written informed consent was obtained from parents of the entire study population.

### Transplant procedures

Following standard protocol for neutropenic patients as per AFBMTC/NIBMT Infection prevention and control measures, all patients were admitted in transplant isolation rooms with laminar flow and HEPA filters. MRSA screening and stool surveillance for vancomycin resistant enterococci (VRE) and carbapenamase resistant enterococci (CRE) were ensured. Antiviral, anti-fungal and pneumocystis jirovecii prophylaxis was administered to all patients. Details of conditioning protocol are given in Table-I.

**Table-I:** Characteristics of Transplant Recipients

Characteristics	VOD(n=40) Mean ± 2 SD	Without VOD (n=345) Mean ± 2 SD	P-Value
Age (months)	40 ± 31	69 ± 38	0.041
***Gender***			0.634
Male	26	227	
Female	14	118	
Bilirubin	16.1 ± 9.5	29.5 ± 85.7	0.324
***Liver Size***			0.723
>5 cm	4	31	
<5 cm	36	314	
Ferritin	2046 ± 1100	2051 ± 1197	0.981
***Pesaro Classification***			0.097
Class I	1	28	
Class II	7	98	
Class III	32	219	
***Fibrosis***			0.000
Mild (Grade 1)	10	345	
Moderate (Grade 2-3)	30		
***Conditioning Protocol***			
Busulphan IV	35	216	
Busulphan Oral;	4	162	0.233
Treosulphan	1	7	0.849
***ABO Mismatch***			0.618
Yes	15	144	
No	25	201	
***Stem Cell Source***			0.158
BMH	35	302	
PBSC	1	29	
BMH+PBSC	2	11	
Boost with BMH/PBSC	2	2	
Second Transplant	0	1	
CD 34 Dose (10^6^/kg)	5.94 ± 2.86	7.70 ± 5.13	0.061
TNC Dose (10^8^/kg)	5.01± 1.38	5.11± 1.40	0.683
Neutrophil Engraftment	14.5 ± 1.82	14.4 ± 4.09	0.847
Platelet Engraftment	31.5 ± 13.7	34.8 ± 88.7	0.834

Neutrophil engraftment was defined as the first day on which absolute neutrophil count (ANC) > 500 cells/microliter was achieved for at least three consecutive days. Platelet engraftment was defined as the first day of achieving platelet count > 20,000/microliter for at least seven days without transfusion support. Transplant related mortality (TRM) was defined as deaths related to transplant.

### VOD: Diagnosis, Risk Classification, Prophylaxis and Management

Patients developing VOD/SOS within 60 days post-transplant were included in the study. VOD/SOS was diagnosed and risk stratified as per EBMT Diagnostic Criteria and was classified as mild, moderate, severe or very severe.^12^ Risk factors for VOD were grouped as: Recipient related: age, Pesaro classification, serum Ferritin, liver size, chelation, fibrosis and Hepatitis B and C status, Transplant related: chemotherapy protocol (dose and routine of administration of alkylating agent, Busulphan) and Donor related: ABO mismatch and gender mismatch.

All patients received VOD/SOS prophylaxis by oral Ursodeoxycholic acid at a dose of 10mg/kg/day and were managed by supportive measures consisting of fluid restriction, diuresis, transfusion support and strict monitoring of intake output, weight gain, change in liver size & abdominal girth. Calcineurin inhibitors and azoles were stopped in all the patients & methylprednisolone was given at a dose of 2mg/kg/day for Graft versus host disease (GVHD) prophylaxis till resolution of VOD/SOS or normalization of bilirubin and ALT. Due to financial constraints only two patients were given defibrotide as a specific therapeutic option, both the patients had mild VOD/SOS and responded within a few days.

### Statistical Analyses

The study was primarily aimed at determining the cumulative incidence and outcome of VOD/SOS in BTM patients and identifying the risk factors associated with it. Incidence of VOD/SOS was calculated using cumulative incidence estimates (%) with a 95% confidence interval (95% CI). Overall survival was calculated using Kaplan Meier estimates with group differences calculated using long rank tests. Frequency and percentage were calculated for categorical variables while Chi-square test was used for quantitative variable. Univariate analysis was used to determine significance of different variables and development of VOD/SOS. A p value of 0.05 or less was considered statistically significant. Statistical analysis was carried out using SPSS version 25.0.

## RESULTS

Transplant characteristics of recipients are shown in Table-I. A total of forty patients out of three hundred and eighty-five BTM patients developed VOD/SOS post HSCT. Median time for development of VOD/SOS was 14 days (range 6-30 days). Cumulative incidence of all grade VOD was 10.39% (95% CI, 7-14). Cumulative incidence of mild, moderate, severe and very severe VOD/SOS was 60% (95% CI 44.6-73.6), 10% (95% CI 3.9-23), 5% (95% CI 1.3-16) and 25% (95% CI 14-40) respectively. 87.5% (35/40) of patients with VOD/SOS had unexplained consumptive and transfusion dependent refractory thrombocytopenia and hepatomegaly, while 62.5% (25/40) had a weight gain of > 5% from baseline and 32.5% (13/40) developed ascites.

### Risk Factors associated with VOD/SOS

Age of the recipient and fibrosis of liver were found to be significantly associated with VOD/ SOS. (p-value 0.04 and 0.000 respectively). Overall age of the recipients was found to be significantly associated with fibrosis. (p value 0.000) with milder fibrosis more in younger age group as compared to older. Univariate analysis of all the risk factors is shown in Table-II.

**Table-II:** Risk factors associated with VOD/SOS

Variables	Total number (N=385)	VOD (N=40)	Hazard Ratio (95% CI)	P Values
***Age group (months)***				
<48	135	3	0.022	0.942
48-84	116	21	0.181	0.552
>84	124	16	0.129	0.671
***Pesaro Classification***				
Class 1	29	1	0.094	0.212
Class 2	107	7	0.062	0.132
Class 3	249	32		
***Ferritin***			0.02	0.527
<2000ng/ml	220	24		
>2000ng/ml	165	16		
***Fibrosis***			0.120	0.000
Mild (Grade 1)	220	10		
Moderate (Grade 2-3)	165	30		
***ABO Mismatch***				
None	331	25	0.035	0.389
Minor	75	9	0.044	0.375
Major	79	6	-	-
***Chelation***			0.029	0.665
Regular	46	2		
Irregular	339	38		
***Sex Mismatch***				
No	186	17	0.022	0.493
Yes	199	23		
***HBV***				
Negative	363	40	0.110	0.483
Positive	22	0		
***HCV***				
Negative	327	36	0.046	0.3
Positive	58	4		
***Liver Size***				
<5	360	35	0.112	0.727
>5	25	5	0.160	0.625
***Conditioning Dose***				
Bu 16mg/kg	222	9	0.209	0.053
Bu 14mg/kg	155	29	0.063	0.562
Tre 14gm/m^2^	8	2	0	
***Conditioning Route***				
I/V Busulphan	216	35	0.161	0.233
Oral Busulphan	162	4	0.026	0.849
Treosulphan	7	1		

### Survival

**Fig.1:**
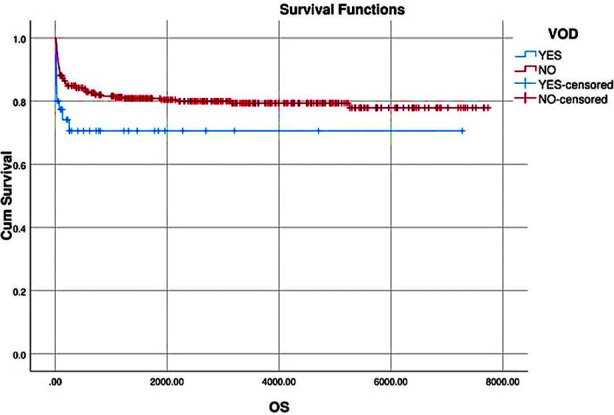
Kaplan Meier curves for OS of BTM with and without VOD/SOS

Out of 40 patients who developed VOD/SOS, eleven died. Nine succumbed to the disease itself, all having very severe VOD/SOS (P value 0.000), while one patient died due to sepsis and one had bleeding complications. Both of these patients had mild VOD/SOS. Cumulative incidence of TRM with and without VOD/SOS was 27.5% (95% CI 16.1-42) and 20.5% (95% CI 16.6-25.1). (P value 0.318). Overall survival of BTM patients post-transplant was 79.4%. OS was significantly associated with development of VOD/SOS. OS was 72.5% for patients with VOD/SOS while OS without VOD/SOS was 80.2% (p value 0.048), (Fig.1).

## DISCUSSION

We report an incidence of 10.39% of all grade VOD/SOS and age of recipient and fibrosis as the significant risk factors. Out of 385 transplant patients, forty developed VOD. Median time from date of transplant till onset of VOD was 14 days (range 6-30). 11 out 40 patients who developed VOD died. Cumulative incidence of Transplant related mortality (TRM) for patients with and VOD was 20.5% (95% CI, 16.6-25.1) vs 27.5% (95% CI, 16.1-42) (p value 0.318) respectively. Among risk factors, age of recipient and fibrosis (p value of 0.04 and 0.000 respectively) were found to be significantly associated with VOD. Although in concordance with the incidence reported by Lai et al from China^2^, incidence is higher as compared to previous studies from our own center and may be due to higher no of cases and longer endpoints. Incidence of VOD/SOS as reported by CIBMTR is 4.9% in HSCT patients^13^ while Coppell JA et al has reported a mean incidence of 13.7%.^14^ A varying prevalence of 0 to 62% has been reported in various studies.^15^

Although there have been studies carried out and reported from our institute in the past on experience of allogeneic HSCT in BTM, there has not been a single study on special emphasis on VOD/SOS in post HSCT BTM patients from a country like Pakistan bearing largest burden of the disease. To the best of our knowledge, this is the largest study on VOD/SOS in BTM patients post Allogeneic HSCT. This study will help to understand one of the most important mortality causing event related to transplant of BTM and hence improve the outcome of disease. The Hashmi et al. in 2004 reported VOD/SOS incidence to be 42.1% (8/19 patients) with VOD/SOS as one of the main causes of mortality.^16^ Ullah K et al. reported VOD/SOS of 2.3-5.1% of various hematological disorders between July 2001 and September 2006.^17^,^18^ Veno-occlusive disease of the liver (VOD) occurred frequently after transplantation, particularly in patients with high-risk disease. Data retrieved from Center for International Blood and Marrow Transplant Research (CIBMTR) on outcome of HLA-matched sibling bone marrow transplantation for β-thalassemia major showed that fifty-eight patients (32%) developed VOD/SOS. Approximately one-third of patients with risk class II and 50% of patients with risk class III features developed VOD. It was also noted that VOD/SOS occurred more frequently in patients with a serum ferritin more than or equal to 2500 μg/L compared with those with a serum ferritin less than 2500 μg/L (52% and 32%, respectively).^19^

Various risk factors have been identified as potential contributors of VOD/SOS including younger age of transplant recipients, Bu and Cy containing conditioning regimens, platelet refractoriness, elevated ferritin levels and use of calcineurin inhibitors.^2^,^12^,^20^ We found that fibrosis was the only risk factor significantly associated with VOD/SOS while Lai et al.^2^ did not report any significant correlation between any risk factors and Cheuk et al found age to be significantly associated with VOD/SOS.^21^ Defibrotide, dalteparin and Lipo-PGE1 and Ursodeoxycholic acid have been widely used as prophylactic regimen to reduce incidence of pediatric VOD/SOS.^2^,^22^,^23^ Ursodeoxycholic acid prophylaxis was used in our patients for prevention of VOD/SOS.

According to reported literature, approximately 25% of VOD/SOS fall into severe category with mortality 75-98%.^24^ While a wait and watch policy may be adopted for mild VOD/SOS cases, moderate, severe and very severe cases inevitably warrant early initiation of treatment and a multi-disciplinary approach including admission to a pediatric intensive care unit (PICU). Specific therapy includes early commencement of defibrotide, ideal time of which is not assessed in a randomized trial so far. However, there have been various studies ascertaining the optimum dose and supporting the efficacy of defibrotide for treatment of severe and very severe VOD/SOS.^24^-^27^ Owing to the non-feasibility of defibrotide, it could be administered only to two patients, both of which had a mild VOD/SOS and recovered. Lai et al reported all patients of VOD/SOS in either mild (59.3%) or moderate (40.7%) category and no case of severe or very severe VOD/SOS. We, on the contrary, found 60% mild, 10% moderate, 5% severe and 25% very severe cases. While none of their patients who developed VOD/SOS died due to the disease itself, nine out of eleven of our patients died due to VOD/SOS, all being very severe. While Lai et al reported three year OS rates of 94.3%, our OS rate till the time of last follow up was 72.5% for patients who developed VOD/SOS. Despite availability of scarce literature on the significance of supportive care therapy in the management of VOD/SOS, we adopted a policy of fluid restriction, diuresis, transfusion support and strict monitoring of intake output, weight gain, change in liver size & abdominal girth in addition to withholding of calcineurin inhibitors and azoles till resolution of VOD/SOS and administration of methyl prednisolone for prevention of GVHD. We were, in fact, able to save the lives of all cases of severe and most of mild and moderate cases of VOD/SOS through strict adherence to supportive measures only.

### Limitations

Due to financial constraints, no patient received defibrotide prophylaxis. Therefore, its efficacy in prophylaxis could not be established. Secondly, defibrotide was used in only two patients and majority of patients were managed conservatively.

## CONCLUSIONS

Incidence of VOD/SOS remains high in patients of BTM post allogeneic HSCT. However, careful patient selection of younger patients and mild grade of fibrosis on USG before transplant may help reduce incidence of VOD/SOS. In our study, we effectively managed most of our patients through supportive measures alone with a favorable outcome and thus their significance cannot be undermined in developing countries like Pakistan where defibrotide cannot be given due to various reasons.

### Authors’ Contribution:

**HN & TK:** Conceived, designed and edited the manuscript.

**MK:** Did Data collection and manuscript writing. She is also responsible for the accuracy or integrity of the work.

**TG & QN:** Did review and final approval of manuscript.

## References

[1] Ahmed S, Ayub M, Naeem M, Nazir FH, Hussain A, Ghilzai D (2021). Thalassemia Patients from Baluchistan in Pakistan Are Infected with Multiple Hepatitis B or C Virus Strains. Am J Trop Med Hyg.

[2] Lai X, Liu L, Zhang Z, Shi L, Yang G, Wu M (2021). Hepatic veno-occlusive disease/sinusoidal obstruction syndrome after hematopoietic stem cell transplantation for thalassemia major:incidence, management, and outcome. Bone Marrow Transplant.

[3] Akhtar IK, Ashraf M, Khalid IU, Hussain M (2016). Surgical outcome of spelenectomy in Thalassemia major in children. Pak J Med Sci.

[4] Corbacioglu S, Carreras E, Ansari M, Balduzzi A, Cesaro S, Dalle JH (2018). Diagnosis and severity criteria for sinusoidal obstruction syndrome/veno-occlusive disease in pediatric patients:a new classification from the European society for blood and marrow transplantation. Bone Marrow Transplant.

[5] Cairo MS, Cooke KR, Lazarus HM, Chao N (2020). Modified diagnostic criteria, grading classification and newly elucidated pathophysiology of hepatic SOS/VOD after haematopoietic cell transplantation. Br J Haematol.

[6] Mahadeo KM, Bajwa R, Abdel-Azim H, Lehmann LE, Duncan C, Zantek N (2020). Diagnosis, grading, and treatment recommendations for children, adolescents, and young adults with sinusoidal obstructive syndrome:an international expert position statement. Lancet Haematol.

[7] Sinusoidal obstruction syndrome/veno-occlusive disease:current situation and perspectives:a position statement from the European Society for Blood and Marrow Transplantation (EBMT) (2015). Bone Marrow Transplant.

[8] Corbacioglu S, Jabbour EJ, Mohty M (2019). Risk factors for development of and progression of hepatic veno-occlusive disease/sinusoidal obstruction syndrome. Biol Blood Marrow Transplant.

[9] Shenoy S, Walters MC, Ngwube A, Soni S, Jacobsohn D, Chaudhury S (2018). Unrelated donor transplantation in children with thalassemia using reduced-intensity conditioning:the URTH trial. Biol Blood Marrow Transplant.

[10] Sabloff M, Chandy M, Wang Z, Logan BR, Ghavamzadeh A, Li CK (2011). HLA-matched sibling bone marrow transplantation for β-thalassemia major. Blood.

[11] Roeker LE, Kim HT, Glotzbecker B, Nageshwar P, Nikiforow S, Koreth J (2019). Early clinical predictors of hepatic veno-occlusive disease/sinusoidal obstruction syndrome after myeloablative stem cell transplantation. Biol Blood Marrow Transplant.

[12] Corbacioglu S, Jabbour EJ, Mohty M (2019). Risk factors for development of and progression of hepatic veno-occlusive disease/sinusoidal obstruction syndrome. Biol Blood Marrow Transplant.

[13] Strouse C, Zhang Y, Zhang MJ, DiGilio A, Pasquini M, Horowitz MM (2018). Risk score for the development of veno-occlusive disease after allogeneic hematopoietic cell transplant. Biol Blood Marrow Transplant.

[14] Coppell JA, Richardson PG, Soiffer R, Martin PL, Kernan NA, Chen A (2010). Hepatic veno-occlusive disease following stem cell transplantation:incidence, clinical course, and outcome. Biol Blood Marrow Transplant.

[15] Carreras E, Diaz-Beya M, Rosinol L, Martinez C, Fernández-Aviles F, Rovira M (2011). The incidence of veno-occlusive disease following allogeneic hematopoietic stem cell transplantation has diminished and the outcome improved over the last decade. Biol Blood Marrow Transplant.

[16] Hashmi KU, Khan B, Ahmed P, Hussain I, Rasul S, Hanif E (2004). Allogeneic Bone Marrow Transplantation in beta-Thalassaemia-Single Centre Study. J Pak Med Assoc.

[17] Ullah K, Ahmed P, Raza S, Satti T, Nisa Q, Mirza S (2007). Allogeneic stem cell transplantation in hematological disorders:single center experience from Pakistan. Transplant Proc.

[18] Ullah K, Shamsi TS, Adil SN (2007). Bone Marrow Transplant Activity-A Country Report from Pakistan. Blood.

[19] Sabloff M, Chandy M, Wang Z, Logan BR, Ghavamzadeh A, Li CK (2011). HLA-matched sibling bone marrow transplantation for β-thalassemia major. Blood.

[20] Mcdonald GB, Sharma P, Matthews DE, Shulman HM, Thomas ED (1984). Venocclusive disease of the liver after bone marrow transplantation:diagnosis, incidence, and predisposing factors. Hepatology.

[21] Cheuk DK, Wang P, Lee TL, Chiang AK, Ha SY, Lau YL (2007). Risk factors and mortality predictors of hepatic veno-occlusive disease after pediatric hematopoietic stem cell transplantation. Bone Marrow Transplant.

[22] Cappelli B, Chiesa R, Evangelio C, Biffi A, Roccia T, Frugnoli I (2009). Absence of VOD in paediatric thalassaemic HSCT recipients using defibrotide prophylaxis and intravenous Busulphan. Br J Haematol.

[23] Zirakzadeh A, Montori V, Imran H, Litzow M, Kumar S (2006). Ursodiol prophylaxis against hepatic veno-occlusive disease in hematopoietic stem cell transplant recipients:A systematic review and meta-analysis. Biol Blood Marrow Transplant.

[24] Bajwa RP, Mahadeo KM, Taragin BH, Dvorak CC, McArthur J, Jeyapalan A (2017). Consensus report by pediatric acute lung injury and sepsis investigators and pediatric blood and marrow transplantation consortium joint working committees:supportive care guidelines for management of veno-occlusive disease in children and adolescents, part 1:focus on investigations, prophylaxis, and specific treatment. Biol Blood Marrow Transplant.

[25] Chopra R, Eaton JD, Grassi A, Potter M, Shaw B, Salat C (2000). Defibrotide for the treatment of hepatic veno-occlusive disease:results of the European compassionate-use study. Br. J. Haematol.

[26] Corbacioglu S, Greil J, Peters C, Wulffraat N, Laws HJ, Dilloo D (2004). Defibrotide in the treatment of children with veno-occlusive disease (VOD):a retrospective multicentre study demonstrates therapeutic efficacy upon early intervention. Bone Marrow Transplant.

[27] Bulley SR, Strahm B, Doyle J, Dupuis LL (2007). Defibrotide for the treatment of hepatic veno-occlusive disease in children. Pediatric Blood Cancer.

